# Low Complexity Equalization of Orthogonal Chirp Division Multiplexing in Doubly-Selective Channels

**DOI:** 10.3390/s20113125

**Published:** 2020-06-01

**Authors:** Xin Wang, Zhe Jiang, Xiao-Hong Shen

**Affiliations:** School of Marine Science and Technology, Northwestern Polytechnical University, Xi’an 710072, China; wangxin1103@mail.nwpu.edu.cn (X.W.); jzh1723@nwpu.edu.cn (Z.J.)

**Keywords:** OCDM, doubly-selective channels, channel equalization, BEM, underwater acoustic communications

## Abstract

Orthogonal Chirp Division Multiplexing (OCDM) is a modulation scheme which outperforms the conventional Orthogonal Frequency Division Multiplexing (OFDM) under frequency selective channels by using chirp subcarriers. However, low complexity equalization algorithms for OCDM based systems under doubly selective channels have not been investigated yet. Moreover, in OCDM, the usage of different phase matrices in modulation will lead to extra storage overhead. In this paper, we investigate an OCDM based modulation scheme termed uniform phase-Orthogonal Chirp Division Multiplexing (UP-OCDM) for high-speed communication over doubly selective channels. With uniform phase matrices equipped, UP-OCDM can reduce the storage requirement of modulation. We also prove that like OCDM, the transform matrix of UP-OCDM is circulant. Based on the circulant transform matrix, we show that the channel matrices in UP-OCDM system over doubly selective channels have special structures that (1) the equivalent frequency-domain channel matrix can be approximated as a band matrix, and (2) the transform domain channel matrix in the framework of the basis expansion model (BEM) is a sum of the product of diagonal and circulant matrices. Based on these special channel structures, two low-complexity equalization algorithms are proposed for UP-OCDM in this paper. The equalization algorithms are based on block LDL${}^{H}$ factorization and iterative matrix inversion, respectively. Numerical simulations are finally proposed to show the performance of UP-OCDM and the validity of the proposed low complexity equalization algorithms. It is shown that when the channel is doubly selective, UP-OCDM and OCDM have similar BER performance, and both of them outperform OFDM. Moreover, the proposed low complexity equalizers for UP-OCDM both show better BER performance than their OFDM counterparts.

## 1. Introduction

In the last two decades, high-speed data communication over dispersive channels has been widely investigated. One of the most successful modulation schemes is orthogonal frequency division multiplexing (OFDM) due to its ability to transmit information symbols on orthogonal subcarriers [1]. However, because the subcarriers used in OFDM are cosine signals, which are relatively sensitive to Doppler spread, when the channel is doubly selective, the performance of OFDM systems will suffer serious degradation.

A chirp signal is a signal whose frequency increases or decreases with time. Due to the good temporal resolution of the autocorrelation function and the insensitivity to Doppler effects, chirp signals are extensively used in radar and sonar applications [2,3,4]. In the areas of communication, chirp spread spectrum (CSS) techniques are widely applied for applications between mobile nodes [5,6]. In order to obtain a higher data rate, a common way is to transmit a bank of chirp signals without interference in one period. Based on this principle, Fractional Fourier Transform-Orthogonal Frequency Division Multiplexing (FrFT-OFDM) is proposed in Reference [7], which is able to generate orthogonal chirp signals via fractional Fourier transform (FrFT). It is shown in Reference [7] that the performance of FrFT-OFDM is better than OFDM over varying Doppler spread factors. However, FrFT-OFDM systems require an estimation of the angle parameter α, which corresponds with the Doppler spread factor. The estimation will lead to computational overhead and is hard to realize in fast varying channels. In Reference [8], a novel multicarrier modulation system called orthogonal chirp division multiplexing (OCDM) has been proposed. It is shown that when the channel is time-invariant, OCDM can achieve better performance with similar peak-to-average power ratio (PAPR) and spectral efficiency when compared with the well studied orthogonal frequency division multiplexing (OFDM) technology [8]. In the meantime, the receiver structure is simpler than FrFT-OFDM because no extra parameter estimation is needed. OCDM has already been applied for optical and underwater communications [9,10,11,12,13,14]. In Reference [9], OCDM is firstly applied for high-speed coherent optical communication, then an underwater acoustic communication system based on the underloaded OCDM system is proposed in Reference [10]. However, in OCDM, the use of different phase matrices might lead to extra storage overhead. More importantly, since the emergence of OCDM is relatively new, most existing research has focused on their performance over frequency-selective channels so far [8,9,13,14]. When the channel is doubly selective, the performance of OCDM systems is mainly investigated in underwater acoustic communication (UAC) applications with experimental channels recorded in field tests [10,11,15]. Phase correcting methods are applied in the proposed UAC systems to deal with Doppler spreads by estimating and compensating the Doppler phase shift in each block, which will lead to extra computational costs. To date, low complexity equalization algorithms for OCDM based systems over doubly selective channels have not been investigated yet.

In this paper, we propose an OCDM based modulation scheme termed uniform phase-Orthogonal Chirp Division Multiplexing (UP-OCDM) for high-speed communication over doubly selective channels. By using uniform phase matrices, UP-OCDM can reduce the storage requirement of OCDM while maintaining the time-frequency diversity gain and high spectral efficiency. Then we prove that like OCDM, the transform matrix of UP-OCDM is circulant. Based on the circulant structure, two low complexity equalization methods are proposed for UP-OCDM over doubly selective channels. The first one is proposed in the frequency domain. We derive that the equivalent frequency-domain channel matrix of UP-OCDM system can be approximated as a band matrix. And the second one is proposed in the framework of the basis expansion model (BEM) [16,17,18,19]. We will show in this paper that the transform domain matrix channel of UP-OCDM can be represented as a sum of the product of several diagonal and circulant matrices. By directly performing the LSQR algorithm [20] on the time-domain receive signal, low complexity iterative equalization is achieved.

This paper is organized as follows. In Section 2, the UP-OCDM signal model, the circulant structure of the transform matrix, and the transmission model over the doubly selective channel is presented. In Section 3, firstly, the minimum mean square error (MMSE) equalizer for UP-OCDM is given. Then based on the circulant structure of the transform matrix, the band MMSE block equalization and the iterative LSQR equalization are proposed, and the complexities are presented. Numerical simulation results are proposed in Section 4. Finally, conclusions are drawn in Section 5.

The notation used in this paper is summarized as follows. Bold upper (lower) letters denote matrices (column vectors); ${\left(\cdot \right)}^{*},{\left(\cdot \right)}^{T}$ and ${\left(\cdot \right)}^{H}$ stand for conjugate, transpose, and Hermitian transpose respectively. ⊙ denotes the element-wise product. We define x[n] as the *n*th entry of the vector x, and X(m,n) as the (m,n)th entry of the matrix X, where all indices are starting from 0. F stands for the N×N unitary DFT matrix, that is, $F\left(p,q\right)=N^{-\frac{1}{2}}e^{-j2\pi \frac{pq}{N}}$; $I_{N}$ refer to the N×N identity matrix; D(x) is denoted as a diagonal matrix created from x.

## 2. System Model

### 2.1. Signal Model of UP-OCDM

In this section, we will introduce the signal model of UP-OCDM first. We consider a bank of baseband continuous chirp signals with chirp rate equaling to $a=-\frac{N}{T^{2}}$, where *N* is the number of subcarriers, and *T* is the symbol duration. Therefore, the chirp signals can be expressed as

(1) $$\begin{array}{cc} c_{k}\left(t\right) & =e^{j\pi a{\left(t-k\frac{T}{N}\right)}^{2}}\\ \; & =e^{-j\pi \frac{N}{T^{2}}{\left(t-k\frac{T}{N}\right)}^{2}},0\leq t<T. \end{array}$$

Notice that the chirp waveforms are mutually orthogonal,

(2) $$\int_{0}^{T}e^{-j\pi \frac{N}{T^{2}}{\left(t-m\frac{T}{N}\right)}^{2}}e^{j\pi \frac{N}{T^{2}}{\left(t-k\frac{T}{N}\right)}^{2}}dt=\delta \left(m-k\right).$$

Thus, similar to the OFDM modulation schemes, the modulated transmission signal is

(3) $$s\left(t\right)=\sum_{k=0}^{N-1}d_{k}c_{k}\left(t\right),0\leq t<T,$$

where $d_{k}$ is the symbol transmitted by the *k*th subcarrier, and is drawn from a finite constellation (e.g., 4-QAM, 16-QAM, 64QAM). We assume a sampling frequency of $f_{s}=\frac{N}{T}$, the baseband discrete signal is

(4) $$s\left(n\right)=\sum_{k=0}^{N-1}d_{k}e^{-j\frac{\pi }{N}{\left(n-k\right)}^{2}},0\leq t<T.$$

Equivalently, the matrix form of (4) is

(5) s=Φd,

where $s={\left[s_{0},s_{1},\ldots ,s_{N-1}\right]}^{T}\in C^{N}$ is the modulated signal, and $d={\left[d_{0},d_{1},\ldots ,d_{N-1}\right]}^{T}\in C^{N}$ is the symbol vector. And

(6) $$\begin{array}{cc} \Phi (m,n) & =e^{-j\frac{\pi }{N}{\left(m-n\right)}^{2}}\\ \; & =e^{-j\pi \frac{m^{2}}{N}}e^{j2\pi \frac{mn}{N}}e^{-j\pi \frac{n^{2}}{N}}. \end{array}$$

Define the phase matrix as

(7) $$\Gamma =D(\left[1,e^{-j\pi \frac{1^{2}}{N},\ldots ,e^{-j\pi \frac{{\left(N-1\right)}^{2}}{N}}}\right]).$$

Therefore, the transform matrix Φ can be rewritten as

(8) $$\Phi =\Gamma F^{H}\Gamma ,$$

where F is the Fourier transform matrix. It is implied in (8) that, with the transmit symbol vector d, the baseband transmit signal s in UP-OCDM can be obtained within the following three steps:

(1) Multiplying the symbol vector d by the phase matrix Γ,
(2) Performing IFFT algorithm,
(3) Multiplying the same phase matrix Γ.

**Figure 1:**
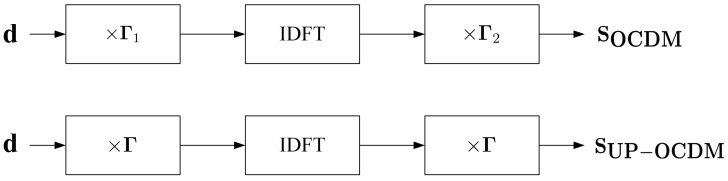
Modulation procedures of the orthogonal chirp division multiplexing (OCDM) (upper) and uniform phase-OCDM (UP-OCDM) (lower).

Note that compared with OCDM, the number of complex parameters to be stored by UP-OCDM for phase matrices is halved of that in OCDM, this is because OCDM needs to multiply two different matrices while UP-OCDM uses two uniform phase matrices denoted as Γ. We will also prove in the next subsection that the transform matrix of UP-OCDM is circulant so that low complexity equalization can be achieved based on this property. Meanwhile, the computation complexities of modulation remain the same in both themes. The comparison between modulation procedures of UP-OCDM and OCDM is given in Figure 1.

Speaking of the spectral efficiency, the bandwidth of the chirp subcarrier in UP-OCDM is $B=N\Delta f=\frac{N}{T}$. Therefore, the analog UP-OCDM signal is likely to occupy a bandwidth of $B+\frac{N}{T^{2}}T=2B$. Whereas, it is proved in Reference [8] that, if each chirp signal has a bandwidth of $B=\frac{N}{T}$, the spectra of each chirp subcarrier can be folded into the baseband from $-\frac{B}{2}$ to $\frac{B}{2}$ with a sampling rate of exactly $f_{s}=B=\frac{N}{T}Hz$. Therefore, by applying up-sampling, the spectrum of UP-OCDM can be fit into the OFDM spectrum, that is, occupying the same bandwidth of *B*.

### 2.2. Circulant Structure of the Transform Matrix

In this subsection, we will discuss the circulant structure of the transform matrix Φ first. With this feature, low complexity equalization algorithms will be proposed in the following sections. Reminding of (6), the (m,n)th element of Φ is

(9) $$\Phi \left(m,n\right)=e^{-j\frac{\pi }{N}{\left(m-n\right)}^{2}}.$$

Define k=m−n and Φ(m−n)=Φ(m,n), we have

(10) Φ(k)=Φ(m,n)=Φ(n,m).

Thus, the transform matrix can be rewritten as

(11) $$\Phi =[\begin{array}{cccc} \Phi \left(0\right) & \Phi \left(1\right) & \cdots & \Phi \left(N-1\right)\\ \Phi \left(1\right) & \Phi \left(0\right) & \cdots & \Phi \left(N-2\right)\\ \vdots & \vdots & \ddots & \vdots \\ \Phi \left(N-1\right) & \Phi \left(N-2\right) & \cdots & \Phi \left(0\right) \end{array}].$$

On the other hand, for any integer L∈[0,N−1],

(12) $$\begin{array}{cc} \Phi (N-L) & =e^{-j\pi \frac{{\left(N-L\right)}^{2}}{N}}\\ \; & =e^{-j\pi \frac{N^{2}-2NL+L^{2}}{N}}\\ \; & =e^{-j\pi N}\times e^{j2\pi L}\times e^{-j\pi \frac{L^{2}}{N}}\\ \; & =e^{-j\pi N}\times e^{-j\pi \frac{L^{2}}{N}}. \end{array}$$

when *N* is odd, $e^{-j\pi N}$ equals to 1, we have

(13) $$\begin{array}{cc} \Phi (N-L) & =e^{-j\pi N}\times e^{-j\pi \frac{L^{2}}{N}}\\ \; & =e^{-j\pi \frac{L^{2}}{N}}\\ \; & =\Phi (L). \end{array}$$

Finally, in UP-OCDM, Φ can be expressed as below and is shown to be a circulant matrix.

(14) $$\Phi =[\begin{array}{cccc} \Phi \left(0\right) & \Phi \left(N-1\right) & \cdots & \Phi \left(1\right)\\ \Phi \left(1\right) & \Phi \left(0\right) & \cdots & \Phi \left(2\right)\\ \vdots & \vdots & \ddots & \vdots \\ \Phi \left(N-1\right) & \Phi \left(N-2\right) & \cdots & \Phi \left(0\right) \end{array}].$$

### 2.3. Transmission Model over Doubly Selective Channels

We consider doubly selective channels in this paper. In order to combat the intersymbol interference (ISI), a cyclic prefix will be added at the beginning of each transmission symbol, the length of the cyclic prefix is chosen so large that exceeds the channel’s maximum delay. After removing the cyclic prefix at the receiver, the received signal can be written as

(15) $$r\left[n\right]=\sum_{l=0}^{L-1}h_{l}\left[n\right]s\left[n-l\right]+w\left[n\right],\;\;\;n=0,1,\ldots ,N-1,$$

where $h_{l}\left[n\right]$ refers to the complex tap gain of the *l*th path at time constant *n*, w[n] is the complex additive white gaussian noise (AWGN) with variance $\sigma_{w}^{2}$, *L* is the channel length and the channel’s maximum delay equals to (L−1)T. Note that s[−l]=s[N−l] due to the existence of cyclic prefix. We assume $L_{cp}=L$ for simple notation in this paper. Equivalently, the transmit-receive relationship (15) can be written as

(16) r=Hs+w,

where $r={\left(r\left[0\right],r\left[1\right],\cdots ,r\left[N-1\right]\right)}^{T}$ is the time-domain receive signal, $s={\left(s\left[0\right],s\left[1\right],\cdots ,s\left[N-1\right]\right)}^{T}$ is the time-domain transmit signal, $w={\left(w\left[0\right],w\left[1\right],\cdots ,w\left[N-1\right]\right)}^{T}$ is the additive white gaussian noise vector in time domain, and H is the time-domain channel matrix.

## 3. Low Complexity Equalizers for UP-OCDM Under Doubly Selective Channels

In this section, based on the transmission model, we propose the MMSE equalization first. Then based on the special circulant structure of Φ, two low complexity equalization algorithms for UP-OCDM are proposed. The first low complexity algorithm is based on LDL${}^{H}$ factorization of the banded approximation of the equivalent channel matrix, and the second one is based on the LSQR algorithm, which is known as an iterative Krylov subspace method. Note that since the transform matrix in OCDM is also circulant, the proposed equalizers can be directly applied for OCDM systems over doubly selective channels.

### 3.1. MMSE Block Equalizer

In this subsection, we propose the time-domain MMSE equalizer for UP-OCDM. With the UP-OCDM input-output relation under doubly selective channel expressed by (15), the linear block MMSE equalization can be written as

(17) $$\begin{array}{cc} {\hat{d}}_{MMSE} & =\Phi^{H}{\hat{s}}_{MMSE}\\ \; & =\Phi^{H}H^{H}{\left({\mathrm{HH}}^{H}+\gamma^{-1}I_{N}\right)}^{-1}r\\ \; & =\Phi^{H}{\left(H^{H}H+\gamma^{-1}I_{N}\right)}^{-1}H^{H}r, \end{array}$$

where γ equals to $\sigma_{d}^{2}/\sigma_{w}^{2}$. Note that the matrix $\left(H^{H}H+\gamma^{-1}I_{N}\right)$ in (17) is Hermitian, and can be solved by LDL${}^{H}$ factorization within $O(N^{3})$ flops [21]. Therefore, the computational complexity of the MMSE block equalizer for UP-OCDM system is $O(N^{3})$, which will be a significant burden for applications with large *N* such as broadcasting applications, or for energy-limited applications such as underwater acoustic communication.

### 3.2. Band MMSE Block Equalizer

In this subsection, we propose an efficient algorithm by exploiting the circulant property of the transform matrix Φ and the band approximation of the Doppler-frequency channel matrix. We find that the equivalent frequency-domain channel matrix can also be approximated as banded in UP-OCDM. With this property, we propose an LDL${}^{H}$ factorization based algorithm to achieve low complexity equalization for UP-OCDM over doubly selective channels.

#### 3.2.1. Band MMSE Equalization Algorithm

At the receiver, we perform an FFT algorithm on the received signal first, and we have

(18) $$\begin{array}{cc} r_{F} & =\mathrm{Fr}\\ \; & =\mathrm{FHs}+\mathrm{Fw}\\ \; & ={\mathrm{FHF}}^{H}F\Phi F^{H}\mathrm{Fd}+w_{F}\\ \; & =H_{E}d_{F}+w_{F}, \end{array}$$

where $H_{E}={\mathrm{FHF}}^{H}F\Phi F^{H}$ is the equivalent channel matrix between $r_{F}$ and $d_{F}$, and $d_{F}=\mathrm{Fd}$ is the transmitted symbols in the frequency domain. Therefore, the MMSE estimate of $d_{F}$ is

(19) $$\begin{array}{cc} {\hat{d}}_{F-MMSE} & =H_{E}^{H}{\left(H_{E}H_{E}^{H}+\gamma^{-1}I_{N}\right)}^{-1}r_{F}, \end{array}$$

where $\gamma =\sigma {{}_{d_{F}}}^{2}/\sigma {{}_{w_{F}}}^{2}=\sigma {{}_{d}}^{2}/\sigma {{}_{w}}^{2}$. It can be seen from (19) that, the computational complexity of obtaining ${\hat{d_{F}}}_{MMSE}$ is also $O(N^{3})$, which is the same as its time-domain counterpart in Section 3.1. Note that $H_{E}$ is the product of ${\mathrm{FHF}}^{H}$ and $F\Phi F^{H}$, where ${\mathrm{FHF}}^{H}$ is the channel frequency response (CFR) matrix and is denoted as $H_{F}={\mathrm{FHF}}^{H}$ in this paper. We will show that by employing the approximately banded structure of the CFR matrix of doubly selective channels and the circulant structure of Φ, $H_{E}$ can be approximated as a banded matrix, so that the complexity of MMSE equalization would be considerably reduced.

Firstly, due to the presence of severe intercarrier interference (ICI) in doubly selective channels, $H_{F}$ is not diagonal as in the time-invariant scenarios; see Reference [22]. Whereas, with a relatively high Doppler spread factor, $H_{F}$ is shown nearly banded, and each diagonal is associated with a discrete Doppler frequency introducing ICI [23]. In this paper, we denote the banded approximation matrix of $H_{F}$ by $H_{B}$. We assume the number of subdiagonals and superdiagonals retained from $H_{F}$ is *Q*, so that the bandwidth of $H_{B}$ is 2Q+1. It is easy to figure out that, the higher *Q* we choose, the smaller approximation error matrix $H_{B}$ has. With a chosen *Q*, $H_{B}$ can be obtained by

(20) $$H_{B}=H_{F}⊙T,$$

where ⊙ is the element-wise product, and T is an N×N banded matrix, with its lower and upper bandwidth both equal to *Q*, and all elements inside the band set as one.

Secondly, with the circulant structure of Φ, it can be easily proved that $F\Phi F^{H}$ is a diagonal matrix with the (k,k)th entry equaling to $e^{j\pi (\frac{k^{2}}{N}-\frac{1}{4})}$. We define $\Lambda =F\Phi F^{H}$, therefore $H_{E}=H_{F}\Lambda$. Note that the modulus of the diagonal elements of Λ equals to 1, therefore, the modulus of the (i,j)th entry of $H_{E}$ equals to that of $H_{F}$, which means $H_{E}$ can also be approximated as a banded matrix with bandwidth 2Q+1. We denote the band approximation of $H_{E}$ as E, so that $E=H_{E}⊙T=H_{B}\Lambda$. Therefore, the matrix $\left(EE^{H}+\gamma^{-1}I_{N}\right)$ in (19) is also a banded matrix with bandwidth 2Q+1, and is denoted as R in this paper. Thus, the inversion of R can be computed by low complexity algorithms. Note that R is symmetric, in this paper, we solve the inversion of R via LDL${}^{H}$ factorization, which is reported below.

In summary, by exploiting the band LDL${}^{H}$ factorization algorithm (Algorithm 1) of R, the MMSE estimate of symbol vector, which is denoted as ${\hat{d}}_{MMSE-BLE}$ can be efficiently obtained in four steps:

(1) Performing FFT algorithm on the received signal r to obtain $r_{F}$;
(2) Constructing the banded matrices E and R. Performing the LDL${}^{H}$ factorization $R={\mathrm{LDL}}^{H}$ to obtain the diagonal matrix D and the triangular matrix L;
(3) Solving the system $\mathrm{Ry}=r_{F}$ by solving firstly the lower-triangular system $\mathrm{Lf}=r_{F}$, secondly the diagonal system Dg=f, and finally the upper-triangular system $L^{H}y=g$;
(4) Obtaining the estimate of symbol vector by ${\hat{d}}_{MMSE-BLE}=F^{H}E^{H}y$.

**Algorithm 1** band LDL${}^{H}$ factorization algorithm

$v=0_{N\times 1};L=I_{N};D=I_{N}$
**for**k=0 to *N* **do** m=max{1,j−2Q};n=min{j+2Q,N}
 **for** i=m to j−1 **do**  $v_{i}=L_{j,i}^{*}R_{i,i};$
 **end for** $v\left(i\right)=R_{i,i}-L_{j,m:j-1}v_{m:j-1}$
 $L_{j,j}-L_{j,m:j-1}$
 $D_{j,j}=v_{j}$
 $L_{j+1:n,j}=\left(R_{j+1:n,m:j}-L_{j+1:n,m:j-1}v_{m:j-1}\right)/vj$

**end for**



#### 3.2.2. Computational Complexity

Speaking of the complexity of the banded block equalizer, we assume the CFR matrix $H_{F}$ is known to the receiver in this paper. Step 1 requires O(NlogN) operations to obtain the receive signal vector in the frequency domain. In step 2, in order to construct R, E=BΛ should be computed first. Since B can be directly constructed from $H_{F}$, so that the computational complexity is that of the product of B and Λ, which is $QN+N-Q^{2}+Q$ complex multiplications (CM). Then we need to compute $EE^{H}$. The computation of ${\left(EE^{H}\right)}_{i,j}$ requires 2Q+1−|i−j| CM and 2Q−|i−j| complex additions (CA). Since $EE^{H}$ is symmetric and banded, by ignoring small terms in the complexity calculation, at most $(2Q^{2}+3Q+1)N$ CM and $(2Q^{2}+Q+1)N$ CA, or $(4Q^{2}+4Q+2)N$ flops in total. Then the band LDL${}^{H}$ algorithm will be applied to obtain D and L. The complexity of the band LDL${}^{H}$ algorithm is totally $(4Q^{2}+6Q)N$ flops [21]. Hence, the computational complexity of step 2 is $\left(8Q^{2}+11Q+3\right)N-Q^{2}+Q$. In step 3, we apply the forward and backward substitution for solving the two triangular systems. The computational complexity of each system is 8QN. Therefore, the total computational complexity of step 3 is (8Q+1)N. In step 4, the computation of $E^{H}y$ needs $\left(2Q+1\right)N-Q^{2}-Q$ CM and $2Q-Q^{2}-Q$ CA, which lead to totally $\left(4Q+1\right)N-2Q^{2}-2Q$ operations. Then we perform the IFFT algorithm on $E^{H}y$, which requires O(NlogN) operations.

Therefore, except for the FFT and IFFT operations in step 1 and step 4, the computational complexity of the band MMSE equalizer is $\left(8Q^{2}+23Q+5\right)N-3Q^{2}-Q$ in total. Because the computational complexity of FFT and IFFT is O(NlogN), the complexity of the band MMSE equalizer is $O(NQ^{2})$.

### 3.3. Iterative LSQR Block Equalizer

In this subsection, we propose an iterative block equalizer for UP-OCDM system based on the circulant structure of the transform matrix and the LSQR algorithm. We will derive that the channel matrix in the transform domain is a sum of the product of several diagonal and circulant matrices under the doubly selective channels modeled by basis expansion models. Based on this structure, the channel matrix between the time-domain receive signal and the transmitted symbol vector can be easily reconstructed, and the transmitted symbol vector could be directly estimated via LSQR algorithm iteratively.

#### 3.3.1. Iterative LSQR Block Equalization Algorithm

We derive the special structure of the transform domain channel matrix first. We assume the channel tap gains are modeled by a basis expansion model, which means each channel tap gain $h_{l}$ is a linear combination of basis functions. With a given set of basis, $h_{l}\left[n\right]$ can be represented as:

(21) $$h_{l}\left[n\right]=\sum_{m=0}^{M-1}c_{l,m}\varphi_{m}\left[n\right],$$

where *M* is the BEM model order, $b_{l,m}$ is the *m*th basis coefficient of the *l*th channel path. Moreover, $\varphi_{m}\left[n\right]$ and $h_{l}\left[n\right]$ denote the *m*th basis function and the channel tap gain of the *l*th path at sampling time $nT_{s}$, respectively. With the increase of *M*, the modeling error can be reduced. Combining the input-output relationship in (15) with the BEM model in (21), the time-domain receive signal r can be expressed as

(22) $$\begin{array}{cc} r & =\sum_{m=0}^{M-1}P_{m}G_{m}s+w\\ \; & =\sum_{m=0}^{M-1}P_{m}G_{m}\Phi d+w\\ \; & =\sum_{m=0}^{M-1}P_{m}\Theta_{m}d+w\\ \; & =Cd+w, \end{array}$$

where $P_{m}=D(\left[\varphi_{m}\left[0\right],\varphi_{m}\left[1\right],\cdots ,\varphi_{m}\left[N-1\right]\right)$ is a diagonal matrix consisted of the BEM basis, $G_{m}$ is a circulant matrix with its first column equaling to ${\left[c_{0,m},c_{1,m},\cdots ,c_{L-1,m}\right]}^{T}$. Note that Φ is also circulant, so that $\Theta_{m}=G_{m}\Phi$ is a circulant matrix. We define $C=\sum_{m=0}^{M-1}P_{m}\Theta_{m}$, which is the transform domain channel matrix between the receive signal r and the transmitted symbol vector d. With the well-known circulant matrix construction method via FFT, it is easy to reconstruct C. After the construction of C, we employ the LSQR algorithm, which is a Krylov subspace method, for the regularized solution of linear systems. Specifically, for a linear system r=Hx, the Krylov subspace for LSQR is spanned as

(23) $$\begin{array}{c} K\left(H^{H}H,H^{H}r,i\right)=Span\left\{H^{H}r,\left(H^{H}H\right)H^{H}r,\ldots ,{\left(H^{H}H\right)}^{\left(i-1\right)}H^{H}r\right\}. \end{array}$$

Essentially, LSQR is an iterative algorithm equivalent to the conjugate gradient method. The main idea of LSQR is to minimize the norm of the residual error in each iteration. It can be concluded from Reference [24] that LSQR is one of the best regularization methods which can achieve the minimum error possible. It will be shown in the simulation section that the LSQR based equalizer is able to achieve performance close to that of the MMSE equalizer when the iteration number is suitably chosen. The details of the LSQR algorithm can be found in Reference [20].

In this paper, we assume the BEM coefficients are already known to the receiver. The first step of the proposed equalization algorithm is to reconstruct the transform domain channel matrix C. It is commonly known that a circulant matrix can be easily constructed by a diagonal matrix. In order to achieve a fast implement, we denote

(24) $$\begin{array}{cc} \Theta_{m} & =F^{H}D_{m}\Lambda F,\\ \; & =F^{H}V_{m}F \end{array}$$

where $V_{m}=D_{m}\Lambda$, and $D_{m}$ is a diagonal matrix whose diagonal elements are the DFT of the BEM coefficients $c_{i,m}$ that zero-padded to length N, i.e.,

(25) $$D_{m}=D\left(F{\left[c_{0,m},c_{1,m},\ldots ,c_{L-1,m},0,\ldots ,0\right]}^{T}\right),$$

and Λ is also diagonal, with $\Lambda \left(k,k\right)=e^{j\pi (\frac{k^{2}}{N}-\frac{1}{4})}$. Therefore, the transform domain channel matrix C can be constructed by

(26) $$\begin{array}{c} C=\sum_{m=0}^{M-1}P_{m}F^{H}V_{m}F. \end{array}$$

Finally, (22) can be directly solved for the transmitted symbols d by LSQR. Specifically, the equalization algorithm takes three steps:

(1) Computing the diagonal matrices $D_{m}$ with the BEM coefficients;
(2) Multiplying $D_{m}$ by the diagonal matrix Λ;
(3) Solving (23) by LSQR algorithm to obtain the symbol vector d;

Therefore, iterative LSQR equalization can directly recover the transmitted signal with time-domain received signal. Furthermore, it is well known that the rate of convergence of LSQR is directly influenced by the condition number of H, which is defined as

(27) $$cond\left(H\right)=\left||H|||\right|H^{-1}\left|\right|.$$

However, Reference [25] shows that the LSQR based systems have a property of semi-convergence, which means that the increase of the iteration times does not always lead to an improvement of inversion performance, this will also be confirmed in our numerical simulation results. On the other hand, it will also be shown in our simulations that the performance of the proposed iterative LSQR equalizer outperforms its OFDM counterparts with an iteration number of 4.

#### 3.3.2. Compational Complexity

Speaking of the complexity of the proposed iterative LSQR equalizer, we assume the BEM coefficients of the doubly selective channels are known to the receiver. Step 1 requires to perform the FFT algorithm for M times, which results in a computation complexity of O(MNlogN) operations. Step 2 requires MN operations to multiply a diagonal matrix by another for M times. In step 3), by using LSQR method, the computational complexity is (2i+1)MNlogN+(10i+3+2Mi+2M)N+14i+1, where *i* denotes the iteration number and M is the order of BEM [25]. Therefore, the total complexity of the LSQR iterative equalizer is O(iNMlogN). A comparison of the computational complexities of the proposed equalizers can be found in Table 1.

## 4. Numerical Simulations

In this subsection, numerical simulation results are provided to show the bit error rate (BER) performance of the proposed UP-OCDM equalization algorithms over doubly-selective channels with exact channel information. We here consider a communication scenario where the symbol duration is *T* = 64 μs for UP-OCDM, OCDM, and OFDM systems. Therefore, the symbol sampling period is T/N = 0.25 μs. All information bits in simulations are encoded with a convolutional code of rate 1/2 before mapping, and are decoded by a Viterbi decoder at the receiver after equalization. We use wide sense stationary uncorrelated scattering (WSSUS) Rayleigh fading channels with Jakes Doppler spectrum in our simulations. The channel memory length is set to L=16 in the simulation and each channel has an exponentially decaying power delay profile of losing 2 dB per tap. The simulated transmit signal is filtered through channels with varying normalized Doppler spread $f_{d}T$. The channel impulse response (CIR) information is assumed known to the receiver. Then the noise which modeled as AWGN is added at the receiver, and the BER is evaluated by $E_{b}/N_{0}$, which means the ratio of bit energy $E_{b}$ to the noise power density $N_{0}$.

### 4.1. Performance with MMSE Equalizer

In order to evaluate the performance of UP-OCDM over doubly selective channels, we compare the performance of the UP-OCDM with OCDM and OFDM methods with the MMSE equalizer proposed in Section 3.1. The BER performance of UP-OCDM, OCDM, and OFDM systems with MMSE block equalizer is proposed over channels with the normalized Doppler frequency selected as 0.1 and 0.3, respectively. Note that these values represent high Doppler spread conditions. For example, for an OFDM system whose carrier frequency is $f_{c}=10$ GHz and the subcarrier spacing is Δf = 20 kHz, the mobile speed would be v=216 km/h and v=648 km/h, respectively. We compare the performance in M-ary QAM with M from 4 to 64.

**Figure 2:**
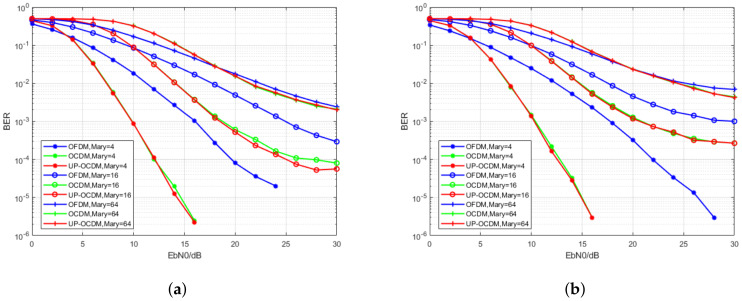
Bit error rate (BER) performance of the UP-OCDM, OCDM, and Orthogonal Frequency Division Multiplexing (OFDM) systems with minimum mean square error (MMSE) equalizers under the 16-ray doubly selective channels with the normalized Doppler spread of 0.1 (**a**) and 0.3 (**b**).

The simulation results are shown in Figure 2a,b. The red curves represent the performance of UP-OCDM, while the blue curves and green curves represent the performance of OCDM and OFDM, respectively. We observe that UP-OCDM and OCDM with MMSE equalizer have similar performance under doubly selective channels. In the meantime, both of them outperform OFDM due to the contributions of diversity gain via using chirped subcarriers. However, it should be noted that, with the modulation level increasing from 4 to 64, the performance of UP-OCDM and OCDM will suffer severer degradation than OFDM. For instance, for the 4-QAM case, the curves of UP-OCDM and OCDM systems intersect the curve of OFDM at about SNR = 5 dB, which means UP-OCDM and OCDM will outperform OFDM when SNR is more than 5 dB, whereas, for the 16-QAM, the intersection is at SNR = 10 dB. This is because high-level modulation formats in UP-OCDM and OCDM are more sensitive to noise. Nonetheless, the BER performance still gets better than OFDM for SNR > 22 dB in the worst scenario, that is, 64-QAM mapping and the normalized Doppler 0.3.

Therefore, with reduced storage requirements, the proposed UP-OCDM shows similar performance as OCDM over doubly selective channels. Meanwhile, due to the exploitation of diversity gain, the performance of both UP-OCDM and OCDM is shown to outperform OFDM under high Doppler spread channels. It should be noted that the performance of OCDM and UP-OCDM might be comparable to the OFDM-FFT spreading system proposed in Reference [26], which is an OFDM based system and also achieves performance improvement via obtaining diversity gain.

### 4.2. Performance with Band MMSE Block Equalization

In this section, the BER performance of UP-OCDM and OFDM with the band MMSE block equalization are compared under the channels with normalized Doppler frequency of 0.1 and 0.3, respectively. We choose a varying approximation *Q* of 2, 4, and 6 in our simulations, which means the bandwidths of the approximated channel matrices are 5, 9, and 13, respectively.

**Figure 3:**
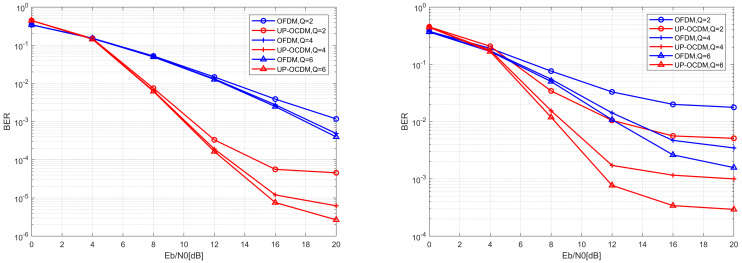
BER performance of the UP-OCDM and OFDM systems with band MMSE block equalizers under different bandwidth *Q* with the normalized Doppler spread of 0.1 (**a**) and 0.3 (**b**).

Figure 3a,b show the BER vs. SNR performance with band MMSE block equalizers for UP-OCDM and OFDM over channels with different Doppler spread. The blue curves represent the performance of OFDM systems, while the red curves represent for UP-OCDM system. From both Figure 3a,b, it can be seen that the proposed block banded equalizers for UP-OCDM outperform its OFDM counterparts. An intuitive explanation is based on the precoded interpretation of UP-OCDM. Recalling Section 3.2, ${\hat{d}}_{MMSE-BLE}=F^{H}E^{H}y$. Note that compared with the OFDM system channel model under the frequency domain, the symbol vector d in UP-OCDM can be seemed as precoded by F, rather than modulated independently on each subcarrier in OFDM. Therefore, the energy of each symbol will be distributed over N subcarriers, and by which diversity gain can be obtained. We can also observe that, with the increase of bandwidth, the performance of both UP-OCDM and OFDM gets better. This is due to the fact that the channel matrix will be approximated more precisely with a bigger bandwidth factor *Q*. However, we can also note from Figure 3b, as the normalized Doppler spread increases, the BER performance of equalizers will suffer degradation. This is because when we are using a banded approximated channel matrix, we ignore the out-of-band ICI. With the same *Q*, the larger $f_{d}T$ will lead to more leakage of the out-of-band ICI, so that the approximation error will increase. Nonetheless, UP-OCDM can still achieve reliable transmissions with Q=4, and the complexity is reduced to $O(NQ^{2})$ from $O(N^{3})$.

### 4.3. Performance with Iterative LSQR Equalization

In this section, the BER performance of the iterative LSQR equalizer for UP-OCDM is proposed. Two equalization methods based on LSQR algorithm for OFDM and respectively proposed in References [25,27], are simulated over the same doubly selective channels with the normalized Doppler spread set as 0.1 and 0.3.

**Figure 4:**
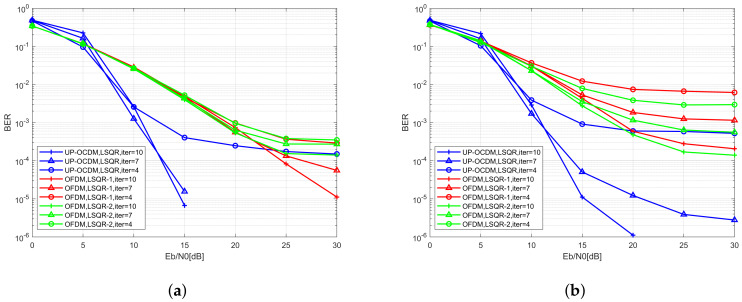
BER performance of the UP-OCDM and OFDM systems with iterative equalization algorithms under different iteration number *i* with the normalized Doppler spread of 0.1 (**a**) and 0.3 (**b**).

The simulation results are given in Figure 4a,b. The blue curve is the BER of UP-OCDM system. The green curve and red curve are the BER of OFDM with the equalizers proposed in References [25,27], respectively. We observe that outstanding performance can be achieved by the iterative LSQR equalizer for UP-OCDM under high Doppler scenarios than the equalizers for OFDM. Moreover, compared with the LSQR based equalization algorithms for OFDM, the complexity of the proposed equalization algorithm is reduced by O(NlogN) because of the circulant structure of transform matrix Φ, which means that the symbols vector d can be directly recovered by applying the LSQR algorithm without an extra step of recovering the symbol vector d from an intermediate variable which is estimated by LSQR first. Moreover, generally, the performance of the proposed algorithm gets better with the increase of iteration numbers, because the inversion error will be smaller with more iterations. However, it should be noticed that, in low SNR regions, a larger iteration number does not always lead to a better BER performance. This is because the proposed LSQR based does not require any statistics information. When the SNR is low, a larger iteration number might cause severer noise amplification and lead to worse BER performance. Nonetheless, the performance of the proposed is better than the similar LSQR based equalizers in OFDM with less computational complexity by O(NlogN). Moreover, compared with the MMSE block equalizer proposed in Section 3.3, the complexity is reduced to O(iNMlogN) from $O(N^{3})$, while maintaining comparable performance.

### 4.4. Performance Comparison of Proposed Equalization Algorithms

In this section, we compare the performance of the proposed low complexity equalization algorithms for UP-OCDM over doubly selective channels with varying normalized Doppler spread set as 0.1 and 0.3. We simulate the band MMSE block equalizer and the iterative LSQR equalizers with varying parameters. For the band MMSE block equalizers, we set the bandwidth *Q* as 2, 4, and 6, while for the iterative LSQR equalizers, we set the iteration number *i* as 1, 4, and 8, respectively. To make a fair comparison, we group the equalizers with various *Q* and *i* into three pairs and confirm the computational complexity of the two low complexity equalizers in each pair to be similar. We set the parameters of the low complexity equalizers in each pair as Q=2/i=1, Q=4/i=4, and Q=6/i=8, respectively. In this paper, we denote the performance of equalizers in the same pair with curves that plotted using the same color.

**Figure 5:**
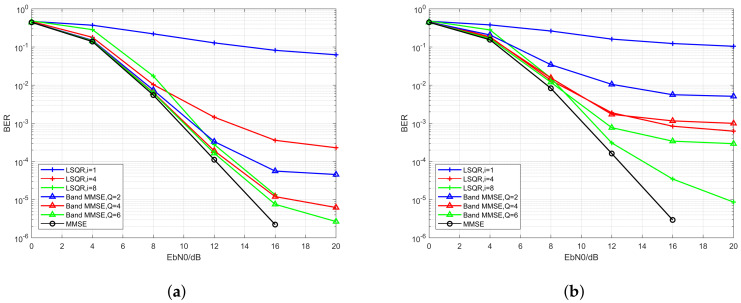
BER performance of the MMSE block equalizer, the band MMSE equalizer, and the iterative LSQR equalizer under different *Q* and *i* with the normalized Doppler spread of 0.1 (**a**) and 0.3 (**b**).

The simulation results are shown in Figure 5a,b. The blue curves denote the pair with the lowest complexities, or the Q=2/i=1 pair. The red curves denote the pair with larger complexities, that is, the Q=4/i=4 pair. Moreover, the green curves represent the pair with Q=6 and i=8, which leads to the largest computational complexities among the three pairs. Besides, the MMSE block equalizers, whose complexities are $O(N^{3})$, are plotted with black curves. From Figure 5a, it can be seen that when the Doppler spread is relatively low, the performance of the band MMSE equalizer can outperform the iterative equalizer with similar computational complexity. Whereas, when it comes to the fast-moving scenario, as shown in Figure 5b, the performance of the band MMSE equalizer will suffer huge degradation, and the performance of the band MMSE equalizers becomes worse than the iterative LSQR equalizers in the second and the third pairs (e.g., the red and the green curves). Moreover, it can be seen from the curves with different colors (e.g., different computational complexity) in both Figure 5a,b that, the performance of the LSQR iterative equalizer can obtain a huge improvement with the increase of computational complexity and might be comparable to the MMSE block equalizer. In other words, the improvement brought by extra computational complexity for the band MMSE equalizer is not so significant as the iterative LSQR equalizers. Moreover, it should be noted that the complexity of the iterative LSQR equalizer is still much lower than the MMSE block equalizer when a relatively large *i* (e.g., i=8 in this section) is used. Finally, we can conclude that, when the Doppler spread is small, or the capacity of computational complexity in the system is severely limited, it might be wise to choose the band MMSE equalizer. On the other hand, when the Doppler spread is large, or the system can bear a relatively higher computational cost, the iterative LSQR equalizer is preferred then.

## 5. Conclusions

In this paper, we proposed an OCDM based modulation scheme termed UP-OCDM system for high rate communication over doubly selective channels. Compared with the OCDM system, UP-OCDM is able to reduce the storage overhead while maintaining the advantages of high spectral efficiency, diversity gaining, and simple receiver structure. Furthermore, with the circulant property of the transform matrix in UP-OCDM, two low complexity equalization algorithms are given, which are based on banded channel approximation and the LSQR algorithm, respectively. Compared with the MMSE equalization algorithm with a computational complexity of $O(N^{3})$, the computational complexities of equalization are reduced to $O(NQ^{2})$ and O(iNMlogN) operations, respectively, where *Q* is the bandwidth parameter, *i* is the iterative number, and *M* is the order of the basis expansion model of the doubly selective channel. Simulation results show that the performance of UP-OCDM and OCDM outperform OFDM, especially in 4QAM and 16QAM scenarios. Besides, the validity of the low complexity methods are confirmed by numerical simulations. Further examination of the impacts of Doppler spread, bandwidth and iteration number on the BER performance is proposed, as well. Finally, a comparison of the proposed low complexity equalization algorithms is given. The results suggest that UP-OCDM has the potential to be equipped for the high-rate wireless communication applications over doubly selective channels.

## Figures and Tables

**Table 1:** Computational Complexity of Proposed Algorithms.

Algorithm	MMSE Equalization	Band MMSE Equalization	Iterative LSQR Equalization
Complexity	$O(N^{3})$	$O(NQ^{2})$	O(iNMlogN)

## Abbreviations

The following abbreviations are used in this manuscript:

OCDM	Orthogonal Chirp Division Multiplexing
UP-OCDM	Uniform Phase-Orthogonal Chirp Division Multiplexing
OFDM	Orthogonal Frequency Division Multiplexing
FrFT	fractional Fourier transform
UAC	Underwater Acoustic Commmunication
BEM	Basis Expansion Mode
MMSE	Minimum Mean Square Error
ISI	intersymbol interference
AWGN	Additive White Gaussian Noise
CIR	Channel Frequency Response
ICI	Intercarrier Interference
CM	Complex Multiplications
CA	Complex Additions
BER	Bit Error Rate
WSSUS	Wide Sense Stationary Uncorrelated Scattering
